# Oral delivery of xenon for cardiovascular protection

**DOI:** 10.1038/s41598-019-50515-3

**Published:** 2019-10-01

**Authors:** Xing Yin, Melanie R. Moody, Valeria Hebert, Melvin E. Klegerman, Yong-Jian Geng, Tammy R. Dugas, David D. McPherson, Hyunggun Kim, Shao-Ling Huang

**Affiliations:** 1 Division of Cardiovascular Medicine, Department of Internal Medicine, The University of Texas Health Science Center at Houston, Houston, Texas 77030 USA; 2 Department of Comparative Biomedical Science, School of Veterinary Medicine, Louisiana State University Health Science Center, Shreveport, Louisiana 71103 USA; 3 Department of Biomechatronic Engineering, Sungkyunkwan University, Suwon, Gyeonggi 16419 Korea

**Keywords:** Cardiovascular diseases, Preventive medicine

## Abstract

Cardiac hypertrophy often causes impairment of cardiac function. Xenon (Xe), a naturally occurring noble gas, is known to provide neurological and myocardial protection without side effects. The conventional method of Xe delivery by inhalation is not feasible on a chronic basis. We have developed an orally deliverable, effective Xe formulation for long-term administration. We employed 2-hydroxypropyl)-β-cyclodextrin (HPCD), which was dissolved in water to increase the Xe concentration in solution. The beneficial effects of long-term oral administration of Xe-enriched solutions on cardiovascular function were evaluated *in vivo*. HPCD increased Xe solubility from 0.22 mM to 0.67 mM (3.8-fold). Aged ApoE knockout mice fed high-fat diet for 6 weeks developed hypertension, and myocardial hypertrophy with impaired cardiac function. Oral Xe prevented this ischemic damage, preserving normal blood pressure, while maintaining normal left ventricular mass and wall thickness. This novel formulation allows for gastrointestinal delivery and cardiovascular stabilization.

## Introduction

Cardiovascular disease (CVD) is a common, and severe medical problem^1^. Attempts to mitigate CVD complications by management of risk factors, include optimization of dietary patterns and components that have cardiac protective effects.

Xenon (Xe) is a cytoprotective gas^2^ with unique advantages such as rapid diffusion^3,4^ with essentially no side effects. Xe cytoprotection involves pleiotypic processes via increases in brain-derived neurotrophic factor (BDNF) and pro-survival proteins such as Bcl-2 that promote cell tolerance to ischemic injury^5^. Xe interacts with the human immune system by modulating inflammatory cytokines such as tumor necrosis factor-α and interleukin-6 in monocytes^6^. Xe helps sustain release of hypoxia inducible factor 1α (HIF-1α) and other modulators of inflammatory processes^7^ such as heme oxygenase-1^8^ and high mobility group protein B1^9^.

Xe has been shown to stabilize and reverse CVD via inhalation delivery system^10–13^. However, inhalation cannot be used for daily Xe delivery. As many cardiovascular diseases result from chronic pathologic progression, there is a need to develop safe orally-administered formulations for long-term use.

Cyclodextrins are cyclic glucopyranose oligosaccharides. Cyclodextrin molecules are cone-shaped, containing a hydrophobic cavity and hydrophilic outer surface. As a result, cyclodextrins can entrap hydrophobic molecules in the internal cavity while dissociating into aqueous liquids. Molecular encapsulation of other gases in cyclodextrins have been previously evaluated^14^. In the present study, we produced a Xe-enriched solution for oral administration through encapsulation of Xe into cyclodextrin.

As beneficial effects have been produced by oral administration of gas-enriched liquids^15–19^, we hypothesize that therapeutic amounts of Xe can be incorporated into a liquid formation for oral delivery. In this study, we developed a formulation of cyclodextrin-encapsulated Xe and demonstrated cardioprotective benefits in an aged ApoE knockout (KO) mouse model.

## Results

### Xe delivery into the circulation by Xe-enriched solution

Gas chromatography-mass spectrometry (GC-MS) demonstrated that the saturation point of Xe in water without a cage vehicle for encapsulation of Xe gas was 0.22 mM. When the cage molecule, (2-hydroxypropyl)-β-cyclodextrin (HPCD) was added, Xe solubility increased from 0.22 mM to 0.67 mM (3.8-fold increase).

A Xe-enriched solution (200 µl) containing 0.67 mM Xe was fed to mice by oral gavage. Ten minutes after oral treatment, GC-MS detected 0.14 ± 0.15 mM Xe in the circulation, demonstrating absorbance of Xe into the peripheral circulation from the stomach.

### Xe-enriched solution stabilized blood pressure

It has been reported that ApoE KO mice fed with high-fat diet are hypertensive due to endothelial disfunction^20,21^. We found that after 6 weeks of normal and high-fat diet, the aged ApoE KO mice demonstrated an increased systolic blood pressure (125 ± 2 mmHg vs. aged ApoE KO mice with normal diet at 98 ± 4 mmHg) (P < 0.05); and increased diastolic blood pressure (88 ± 4 mmHg vs. ApoE KO mice with normal diet at 73 ± 2 mmHg) (P < 0.05). The high-fat diet fed aged ApoE KO mice treated with the vehicle-only solution for 6 weeks has similar systolic (121 ± 5 vs. 125 ± 2 mmHg, P > 0.05) and diastolic (91 ± 1 vs. 88 ± 4 mmHg, P > 0.05) blood pressures compared to those fed with high-fat diet but no treatment. High-fat diet fed aged ApoE KO mice with Xe-enriched solution treatment for 6 weeks had lower systolic (94 ± 2 mmHg vs. 125 ± 2 mmHg, P < 0.05) and diastolic values (77 ± 2 mmHg vs. 98 ± 4 mmHg, P < 0.05) compared to high-fat diet fed ApoE KO mice (Table 1).

**Table 1:** Systolic/diastolic blood pressure in the WT and ApoE KO mice pretreated with or without Xe-enriched solution (200 µl administrated by gavage, once a day for 6 weeks).

	ApoE KO + Normal diet (n = 21)	ApoE KO + High-fat diet (n = 5)	ApoE KO + High-fat diet + Vehicle (n = 5)	ApoE KO + High-fat diet + Xe-enriched solution (n = 5)
Systolic BP (mmHg)	98 ± 4	125 ± 2*	121 ± 5*	94 ± 2^§^
Diastolic BP (mmHg)	73 ± 2	88 ± 4*	91 ± 1*	77 ± 2^§^

Data are expressed as the mean ± S.E.M.

^*^p < 0.05 compared to ApoE KO + Normal diet; ^§^p < 0.05 compared to ApoE KO + High-fat diet + vehicle.

### Xe-enriched solution prevented left ventricular hypertrophy

**Figure 1:**
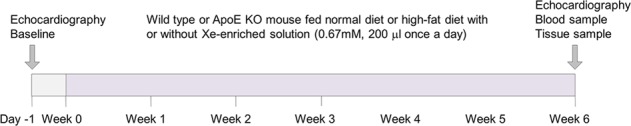
Experiment animal studies. Echocardiography was performed before high-fat diet and treatment. After feeding normal or high-fat diet with or without Xe-enriched solution for 6 weeks, the WT or ApoE KO mouse were undergone echocardiography, blood pressure measurement, and blood/tissue sample test.

**Figure 2:**
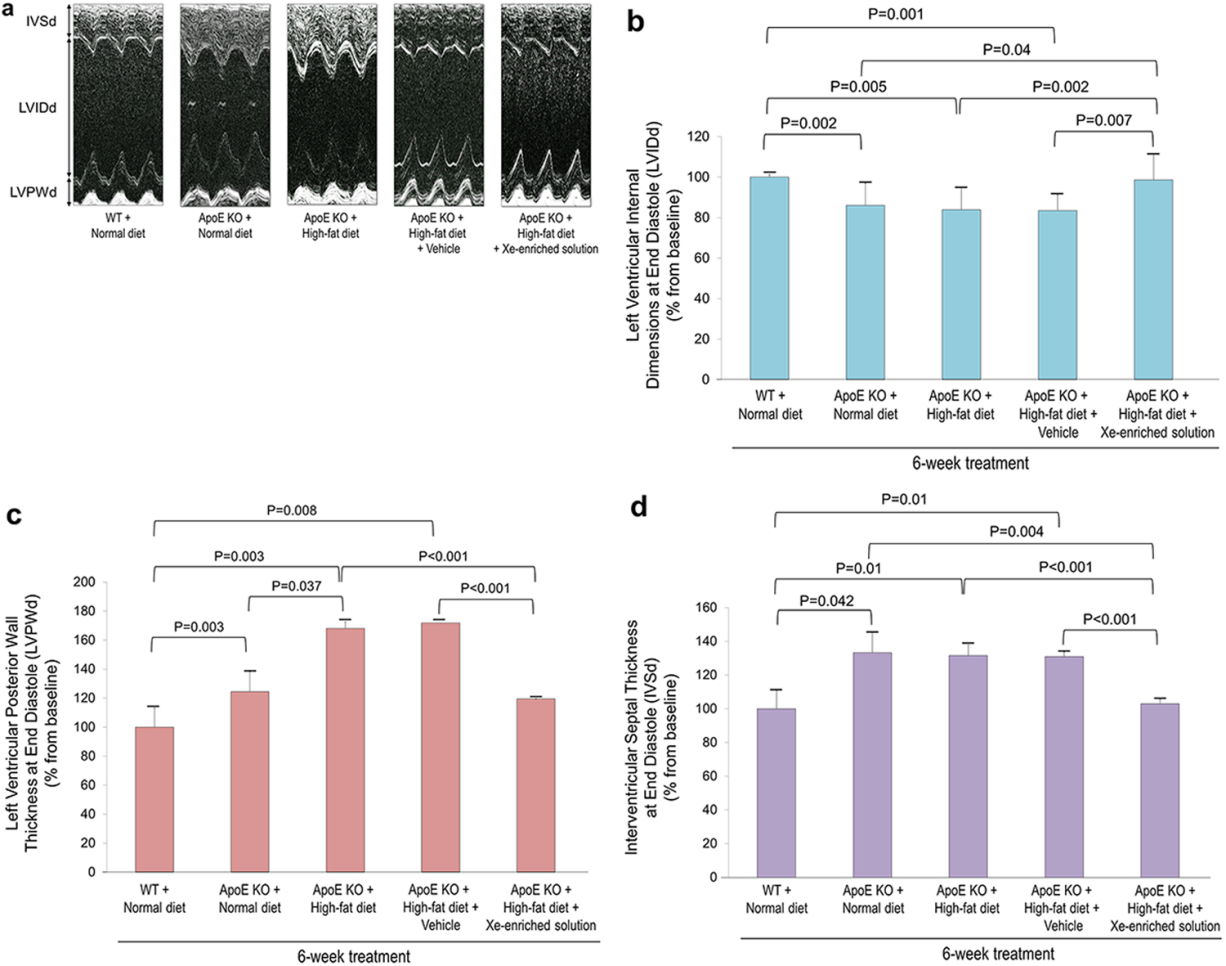
Cardiac dimensions in response to oral Xe treatment (200 µl administrated by gavage, once a day for 6 weeks) in WT and Apo E KO mice fed with/without high-fat diet. (**a**) Representative transthoracic M-mode echocardiographic imaging of the left ventricle of WT and ApoE KO mice with or without treatment, (**b**) LV internal dimensions in diastole (LVIDd), (**c**) left ventricular posterior wall (LVPW) dimensions in diastole (LVPWd), and (**d**) interventricular septal thickness in diastole (IVSd). Data are presented in 6 groups as the percentage change from baseline measurements obtained prior to dietary intervention. WT mice fed with normal diet from baseline (n = 5); ApoE KO mice fed with normal diet from baseline (n = 5); ApoE KO mice fed with high-fat diet from baseline (n = 14); ApoE KO mice fed with high-fat diet and gavaged 200 µl HPCD (5 mg/ml) of vehicle (n = 25). ApoE KO + high-fat diet + Xe-enriched solution (n = 12). Data are expressed as the mean ± S.E.M.

Cardiac hypertrophy is associated with hypertension. Cardiac hypertrophy has been seen in aged ApoE KO mice fed with high-fat diet due to increased afterload from hypertension. We evaluated changes in cardiac morphology following high-fat diet for 6 weeks with or without Xe-enriched solution treatment (Fig. 1). Figure 2 shows representative M-mode echocardiographic images (Fig. 2a) and cardiac dimensions (Fig. 2b–d). Figure 2b demonstrates the left ventricular internal dimensions at end diastole (LVIDd). Smaller LVIDd was found in the ApoE KO mice fed a normal diet (84 ± 2%), high-fat diet (84 ± 3%), and high-fat diet along with vehicle-only (i.e., HPCD) solution (84 ± 2%) than the wild-type (WT) fed a normal diet (100%). As shown in Fig. 2c, ApoE KO mice fed high-fat diet had greater left ventricular posterior wall dimensions at end diastole (LVPWd) (168 ± 13%) than the ApoE KO mice fed normal diet (124 ± 3, P = 0.037), and control WT mice fed normal diet (100%). Drinking Xe-enriched solution with the high-fat diet led to lower LVPWd (120 ± 5%, P < 0.001) compared to those given vehicle-only solution (172 ± 14%) and was close to the ApoE KO mice fed normal diet. These data suggest lack of hypertrophy when Xe-enriched solution was given. Interventricular septal thickness at end diastole (IVSd) is shown in Fig. 2d. Similarly, ApoE KO mice groups fed normal diet (139 ± 13%), high-fat diet (137 ± 7%), and high-fat diet along with vehicle-only solution (136 ± 3%) had greater IVSd than WT control mice fed a normal diet (100%). Lower IVSd (107 ± 3%, P < 0.001) was observed in the mice fed with Xe-enriched solution, again suggesting lack of hypertrophy when Xe-enriched solution was given. Changes in cardiac morphology at systole are summarized in Table 2.

**Table 2:** Systolic cardiac function and cardiac morphology in response to oral Xe-enriched solution (200 µl administrated by gavage, once a day for 6 weeks) treatment.

	LV Vol; d (µl)	LV Vol; s (µl)	IVS; s (mm)	LVID; s (mm)	LVPW; s (mm)	Heart Rate (BPM)
WT baseline	77.5 ± 7.83	30.55 ± 5.34	1.26 ± 0.09	2.86 ± 0.12	1.11 ± 0.08	360 ± 11
ApoE KO baseline	57.8 ± 4.05*	27.99 ± 3.46	1.28 ± 0.07	2.65 ± 0.11	1.13 ± 0.04	439 ± 9*
WT + Normal diet	71.1 ± 2.96	29.67 ± 3.38	1.18 ± 0.07	2.66 ± 0.08	1.18 ± 0.07	377 ± 15
ApoE KO + Normal diet	54.3 ± 5.80*	26.85 ± 2.83	1.32 ± 0.10	2.42 ± 0.14	1.20 ± 0.07	428 ± 19*
ApoE KO + High-fat diet	47.7 ± 5.28*	21.86 ± 4.32	1.42 ± 0.13	2.31 ± 0.11	1.49 ± 0.08	456 ± 15*
ApoE KO + High-fat diet + Vehicle	45.4 ± 4.12*	26.78 ± 3.97	1.43 ± 0.09	2.46 ± 0.13	1.43 ± 0.06	469 ± 19*
ApoE KO + High-fat diet + Xe-enriched solution	58.6 ± 2.90^§§^	22.37 ± 3.94	1.41 ± 0.06	2.24 ± 0.16	1.43 ± 0.09	402 ± 15^§^

Data are expressed as the mean ± S.E.M.

^*^p < 0.05, **p < 0.01, compared to WT baseline and WT + Normal diet, respectively; ^§^p < 0.05, ^§§^p < 0.01, compared to ApoE KO + High-fat diet + Vehicle.

### Xe-enriched solution reduced heart-to-body weight ratio

**Figure 3:**
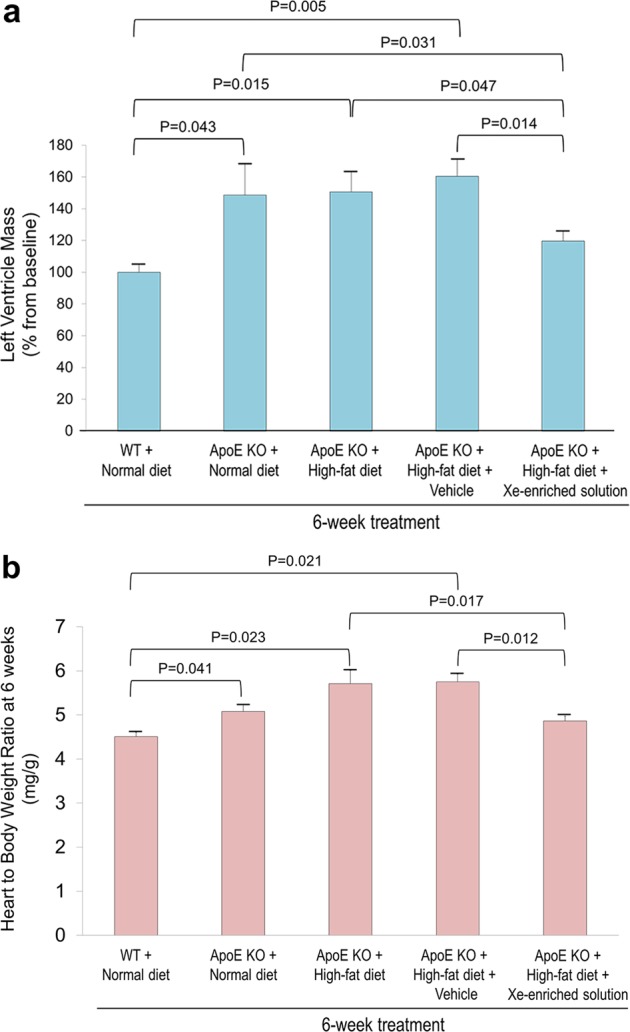
Effect of oral Xe-enriched solution on cardiac hypertrophy. (**a**) LV mass of change in WT and ApoE KO mice fed normal and high-fat diets with and without vehicle-only solution and Xe-enriched solution from baseline to 6 weeks of treatment. (**b**) Ratios of heart weight to body weight after 6 weeks of treatment. WT mice fed with normal diet from baseline (n = 5); ApoE KO mice fed with normal diet from baseline (n = 5); ApoE KO mice fed with high-fat diet from baseline(n = 14); ApoE KO mice fed with high-fat diet and gavaged 200 µl HPCD (5 mg/ml) of vehicle (n = 25). ApoE KO + high-fat diet + Xe-enriched solution (n = 12). ApoE KO mice were administrated Xe gavage (200 µl, once a day). Data are expressed as the mean ± S.E.M.

After 6 weeks, increased left ventricular (LV) mass was observed in the ApoE KO mice fed normal (148 ± 5%) or high-fat (151 ± 13%) diets (Fig. 3a). While vehicle-only solution also showed LV mass increase (160 ± 11%), administration of Xe-enriched solution resulted in a smaller LV mass increase (120 ± 6%, P = 0.014 vs. vehicle-only solution). Figure 3b shows the heart to body weight ratios (mg heart weight/g body weight). Compared to the WT control mice fed normal diet (4.5 ± 0.06), the ApoE KO mice fed either regular or high-fat diets had increased heart-to-body weight ratios (5.1 ± 0.04 and 5.7 ± 0.12, respectively). The Xe-enriched solution stabilized heart-to-body weight ratio close to controls (4.9 ± 0.08, P = 0.94 vs. WT).

### Xe-enriched solution improved cardiac function

**Figure 4:**
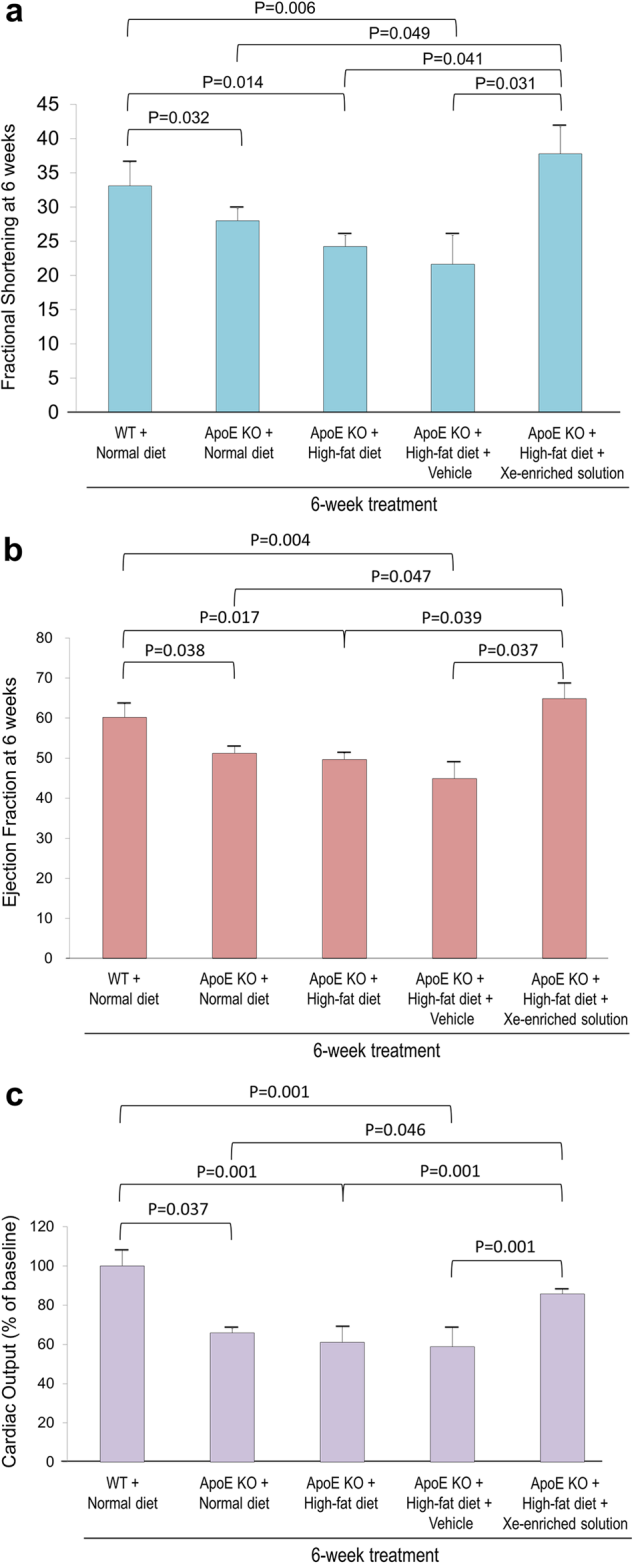
Cardiac function in response to oral Xe treatment. (**a**) Fractional shortening, (**b**) ejection fraction, and (**c**) cardiac output obtained from transthoracic echocardiographic M-mode images of the WT and ApoE KO mice fed normal and high-fat diets with and without vehicle-only solution and Xe-enriched solution (200 µl administrated by gavage, once a day for 6 weeks). Cardiac output in the WT at 6 weeks was used as 100%. Cardiac output data for the other groups are expressed as percentage when compared to WT. Data are expressed as the mean ± S.E.M. and presented as the percentage changes from baseline compared to measurements obtained prior to dietary intervention.

Fractional shortening (FS), ejection fraction (EF), and cardiac output (CO) were measured using M-mode echocardiography (Fig. 4). After 6 weeks of high-fat diet, ApoE KO mice had reduced FS (24 ± 2%) (Fig. 4a), reduced EF (50 ± 4%) (Fig. 4b), and reduced CO (61 ± 3%) (Fig. 4c) when compared with WT control mice fed normal diet. Daily administration of vehicle-solution only did not improve cardiac function in mice fed high-fat diet. However, daily administration of Xe-enriched solution stabilized cardiac function.

### Protection from myocyte dysfunction

**Figure 5:**
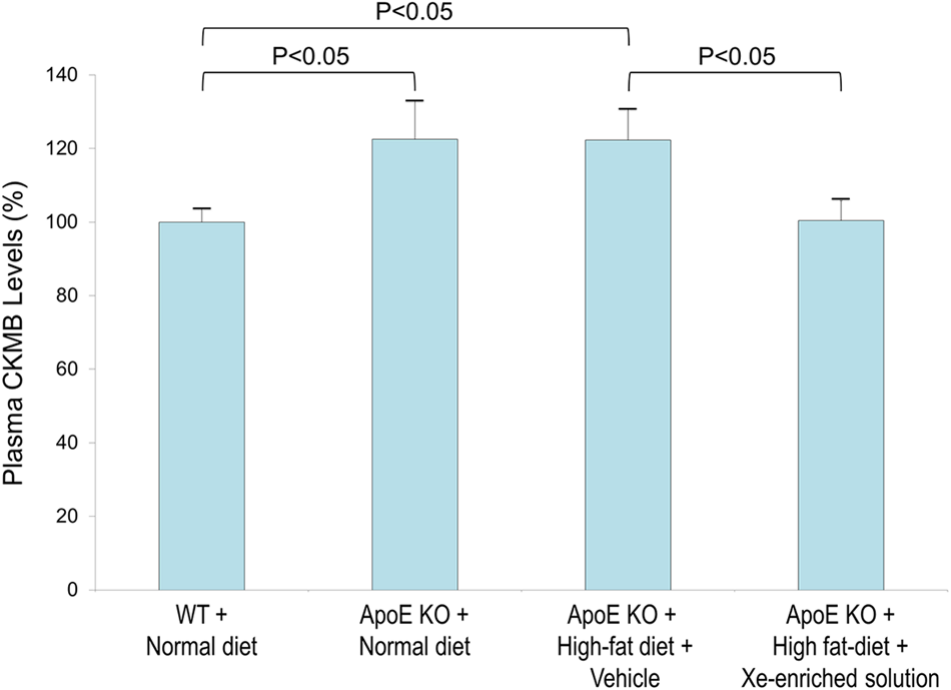
Effect of oral Xe-enriched solution on plasma CKMB levels. Plasma CKMB levels from WT and ApoE KO mice fed normal and high-fat diets with and without control solution (Vehicle) and Xe-enriched solution (200 µl administrated by gavage, once a day for 6 weeks). Data are expressed as the mean ± S.E.M. CKMB levels of WT are expressed as baseline (i.e., 100%). CKMB of the other groups are expressed as percentage when compared to WT.

To evaluate whether Xe-enriched solution can protect the heart from myocyte dysfunction, blood creatine kinase (CK) levels were measured (Fig. 5). Creatine kinase-MB (CKMB), an enzyme responsible for transferring a phosphate group from ATP to creatine, is a sensitive and specific measure of myocardial ischemia^22^. Plasma CKMB levels were measured using ELISA. Increased plasma CKMB levels were found in the ApoE KO mice fed normal diet compared to WT control mice (123 ± 11% vs 100 ± 5%) and ApoE KO mice fed high-fat diet (122 ± 8%). Administration of Xe-enriched solution demonstrated CKMB levels (100 ± 5%) similar to the controls.

### Effect of Xe on BH4/BH2 ratio

Nitric oxide (NO) production plays an important role in endothelial dysfunction and initiation and progression of atherosclerosis. It is known that enzymatic “coupling” of endothelial nitric oxide synthase (coupled eNOS) plays a key role in maintaining endothelial function. The coupling of eNOS is maintained by the balance of Tetrahyrobiopterin (BH4), a required cofactor for eNOS coupling, and 7,8-dihydrobiopterin (BH2), which inactive for NOS cofactor function and compete with BH4 for NOS binding^23^.

**Figure 6:**
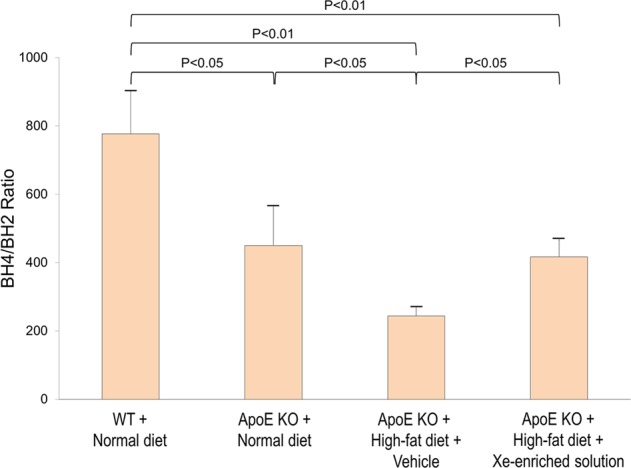
HPLC analysis of BH4/BH2 ratios in the hearts of the WT and ApoE KO mice fed normal and high-fat diets with and without vehicle-only solution and Xe-enriched solution. Data are expressed as the mean ± S.E.M.

Tetrahydrobiopterin (BH4) and dihydrobiopterin (BH2) were measured using high-performance liquid chromatography (HPLC), and the ratio of BH4 to BH2 calculated (Fig. 6). A normal BH4/BH2 ratio (777 ± 126%) was found in the WT mice fed normal diet. The BH4/BH2 ratio was markedly lower in the ApoE KO mice fed normal diet (450 ± 116%) and further decreased in ApoE KO mice fed high-fat diet (244 ± 27%). Mice administrated Xe-enriched solution had higher BH4/BH2 ratios (417 ± 50%) closer to that of ApoE KO mice fed normal diets. This indicated that Xe-enriched solution can correct high-fat diet induced BH4 to BH2 changes.

### Effect of Xe on protein expression *in vivo* and *in vitro*

**Figure 7:**
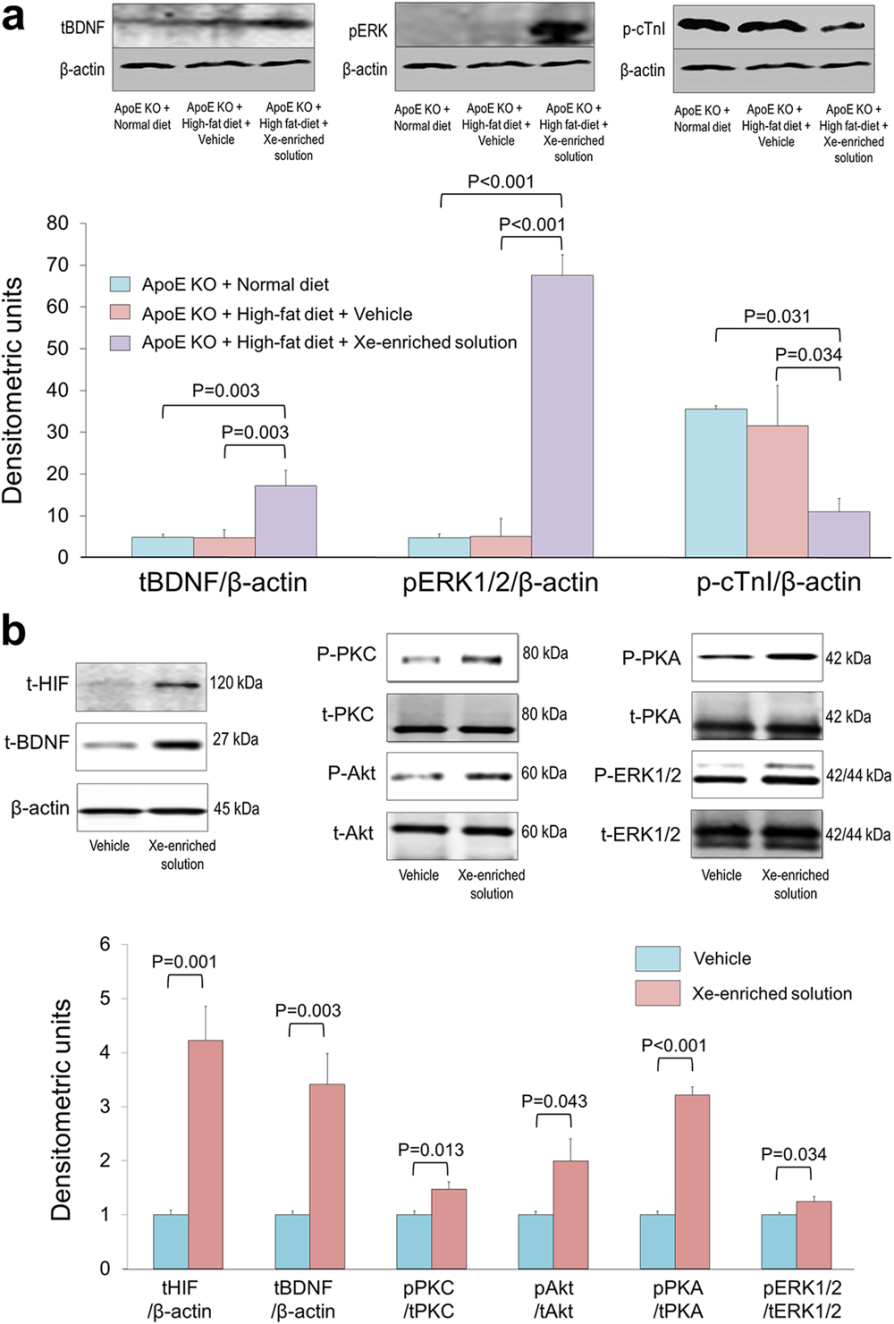
Western blot demonstrating effects of Xe on expression of signaling molecules (**a**) in the cardiac tissue after oral Xe-enriched solution (200 µl administrated by gavage, once a day for 6 weeks) *in vivo*; and (**b**) of cultured neonatal cardiomyocytes with/without 0.22 mM Xe in response to hypoxic stimulation for 24 hours. Data are expressed as the mean ± S.E.M.

To confirm the effect of Xe on myocardial function, cardiac troponin I (cTnI)^22^ and hypoxic stimuli signaling molecules were measured using the western blot (Fig. 7a). The ApoE KO mice fed either normal or high-fat diets showed high cTnI phosphorylation. Xe treatment increased total BDNF expression, and increased ERK1/2 phosphorylation and decreased cTnI phosphorylation in the ApoE KO mice fed high-fat diet compared with the ApoE KO mice fed high-fat diet or high-fat diet with vehicle-only solution. These suggests that Xe help protect myocyte function.

To investigate the mechanism of Xe effect, expression of total- and phospho- hypoxia-inducible factor 1 alpha (HIF-1α), BDNF, cardiac troponin I (cTnI), phospho- protein kinase C (PKC), total- and phospho- protein kinase B (Akt), total- and phospho- protein kinase A (PKA), total and phospho-extracellular signal-regulated kinase 1/2 (ERK1/2) were measured in cultured newborn mouse ventricular myocytes subjected to hypoxia and pre-treated with either vehicle-only or Xe-enriched solution. Figure 7b demonstrates that Xe-enriched solution treatment led to higher total HIF (4.2 ± 0.63 *vs* 1.0 ± 0.09, P = 0.001), BDNF (3.4 ± 0.56 *vs* 1.0 ± 0.07, P = 0.003), PKC (1.5 ± 0.13 *vs* 1.0 ± 0.07, P = 0.013), phosphorylated Akt (2.0 ± 0.41 *vs* 1.0 ± 0.061, P = 0.003), PKA (3.2 ± 0.15 *vs* 1.0 ± 0.06, P < 0.0001), and ERK1/2 (1.3 ± 0.08 vs 1.0 ± 0.04, P = 0.034) levels compared to myocytes treated with vehicle-only solution.

## Discussion

In this study, we evaluated the cardiovascular effects of oral Xe supplementation in ApoE KO mice fed with a high-fat diet and investigated potential pathologies for Xe cardiomyocyte protection. We demonstrated that Xe can be absorbed from the gastrointestinal system into the circulation. When dissolved in solution, Xe acts like a small hydrophobic molecule that can pass through the gastric barrier. *In-vivo* data indicated that daily administration of a Xe-enriched solution for 6 weeks prevented myocyte dysfunction and stabilized blood pressure in transgenic mice prone to these conditions. These findings demonstrate that oral administration of a Xe-enriched solution can be a promising nutraceutical strategy for cardiovascular protection.

ApoE KO mice fed with high-fat diet have been widely used to study the pathogenesis of cardiovascular disease. The ApoE-deficient mouse is considered a translational model of human atherosclerosis. We employed 10 month-old animals and fed them a high-fat diet to induce hypertension and noted development of cardiac hypertrophy. Others have demonstrated that aged ApoE KO mice generate cardiac hypertrophy under these conditions^24^. This felt to be due to the increased aortic stiffening and increased cardiac afterload in the aged ApoE KO mouse^25^. We divided the aged ApoE KO mice into groups of normal diet, high-fat diet, high-fat diet with vehicle, and high-fat diet with Xe-enriched solution. We used the WT as controls only for a model not having cardiovascular changes. We compared our ApoE KO mice with WT. We also compared ApoE KO mice with and without high-fat diet. We compared treatment with vehicle and no treatment. After six weeks of high-fat diet, we found that the animals developed hypertension, cardiac hypertrophy, and changes in cardiac function. Although we chose 6 weeks, these experiments could go on longer with further changes. In general, six weeks in these mice equal to one and half years in human.

Of note, several indicators, including increased left ventricular posterior wall dimensions in diastole, increased interventricular septal thickness, increased left ventricular mass, and increased heart weight/body weight ratio showed cardiac hypertrophy in the ApoE KO mice fed high-fat diet for 6 weeks.

When evaluating the Xe treatment effect, we used the heart weight/body weight ratio. Cardiac hypertrophy assessment using heart weight/body weight ratio has been reported as another indicator of cardiac hypertrophy^26,27^. As an aside, we also observed that administration of Xe-enriched solution for six weeks did not affect total body weight (data are not shown here).

**Figure 8:**
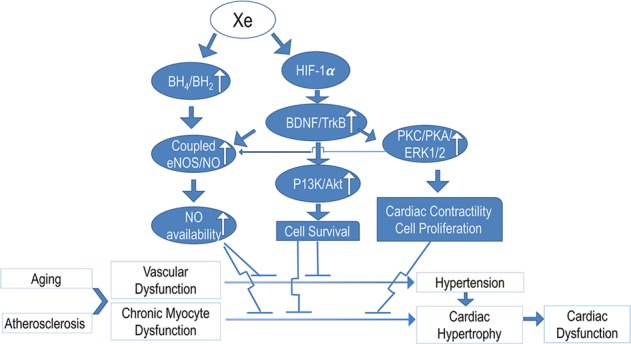
Scheme illustrating the hypothetical mechanism of Xe on stabilizing blood pressure and preventing cardiac hypertrophy in the aged ApoE KO mouse. Tetrahydrobiopterin (BH4); 7, 8-dihydrobiopterin (BH2); Endothelial nitric oxide synthase (eNOS); Nitric oxide (NO); Brain-derived neurotrophic factor (BDNF); Tropomyosin receptor kinase B (TrkB); Phosphatidylinositol 3-kinase (PI3K); Protein kinase B (Akt); Protein kinase C (PKC); Protein kinase A (PKA); Extracellular signal-regulated protein kinases 1 and 2 (ERK1/2).

The mechanism of Xe prevention of cardiac hypertrophy progression in high-fat diet may be related to its effect on stabilizing blood pressure (Fig. 8). One of the primary consequences of atherosclerotic progression is an increase in the stiffness of the aorta and major arteries, leading to hypertension. Endothelial dysfunction is characterized by induction of endothelial NOS (eNOS) uncoupling, which has been observed in ApoE KO mice^28,29^. Uncoupled eNOS produces superoxides, rather than NO, which cause vascular damage. The BH4/BH2 ratio has been identified as a critical molecular mechanism of eNOS uncoupling,^29,30^. BH4 supplementation increases coupled eNOS/NO and improves vascular function^31–33^. We found that Xe administration affects BH4/BH2, a critical regulator of vascular tone. HPLC analysis demonstrated that this was mainly caused by reduction BH2. As BH2 is generated by BH4 oxidation^28,31^, we speculate that Xe affects BH4/BH2 by preventing oxidation of BH4 to BH2. Assessment of several indicators, including creatine kinase-MB (CK-MB) and cardiac troponin I (cTnI) showed that aged ApoE KO mice develop chronic myocyte dysfunction after feeding high-fat diet. Appearance of elevated CK-MB levels in serum is highly specific and sensitive for chronic myocyte injury. cTnI, as a marker of myocyte injury, is more specific and sensitive than CK-MB. Due to its sensitivity, cTnI may be elevated prior to CK-MB changes, allowing for the detection of early myocyte injury^34,35^. In this study, we used CK-MB and cTnI to determine whether there is a chronic myocyte injury in the aged ApoE mouse model fed with high-fat diet. Increased cTnI phosphorylation and plasma CK-MB levels were noted. The Xe-enriched solution preserved normal phosphorylation with lower CK-MB levels - presumably stabilizing myocyte function.

The mechanism by which Xe protects cardiomyocytes from injury may involve regulation of signaling pathway components through multiple pathways. First, Xe upregulates the expression of the total BDNF and downstream Akt, PKA, PKC, and phosphorylated ERK1/2. Secondly, Xe downregulates cTnI phosphorylation and plasma CK-MB levels. Thirdly, Xe up-regulates HIF-1 expression. The latter plays an important role in adaptive responses to hypoxia through a variety of cell processes (from glycolysis to angiogenesis). HIF target genes can also mediate protection against hypoxic damage themselves (Fig. 8).

The important findings of this study are:

1. We are the first to find that this formulation for Xe oral administration can show Xe absorption through the gastrointestinal system into the circulation.
2. We found that Xe stabilized blood pressure, reduced cardiac hypertrophy, and improved cardiac function.
3. We demonstrated a potential mechanism of Xe blood pressure lowing may be related to regulation of coupled eNOS/NO.
4. Xe has low side-effects. It can be taken for a long-time. This type of preparation given orally for a long period of time in low dose could slow the progression of cardiovascular disease.

## Limitations

This study employed the heart/body weight ratio as one of many factors to evaluate cardiac hypertrophy. However, fluctuation in body weight may occur with aging, which may result in impairing the reliability of body weight as a reference for normalizing the heart weight. Another approach to utilize the tibial length to normalize the heart weight can be considered to overcome the potential inaccuracy of the use of the heart/body weight ratio^36^.

Diastolic LV dysfunction observed in this study represents concentric remodeling secondary to hypertension induced by aging and atherosclerosis. We found that systolic LV function in the aged ApoE KO mice did not differ from that in the control mice. The ApoE KO mouse model spontaneously develops hypercholesterolemia and atherosclerotic vascular lesions. Impaired cardiac function in ApoE KO mice is closely related to aging and a Western-type diet. and one of the major limitations of the ApoE KO mouse model is the infrequency of coronary plaques and acute myocardium infarction^24^.

Future studies to compare groups of WT mice with high-fat diet and Xe intervention would allow to investigate the possible effect of such a low Xe concentration on normal conditions and provide valuable information for development of drug application.

## Conclusion

We have developed a Xe formulation that can be orally administrated and demonstrates multiple protective cardiovascular effects. We have demonstrated that Xe can be transported from the gastrointestinal system into the circulation. Thus, this Xe formulation allows for gastrointestinal delivery with cardiovascular stabilization. Further studies on the pharmacokinetics/pharmacodynamics (PK/PD) and toxicity of this formulation would allow translation toward clinical trials and therapeutic use. Further studies of extended dietary regimens and Xe treatment in both WT and ApoE KO mice are needed to evaluate the long-term effects of such a Xe-enriched solution.

## Methods

### Preparation of Xe-enriched solution

The solubility of Xe in water is 0.097 ml/ml. To increase Xe solubility, a Xe-enriched solution was prepared by encapsulating Xe gas (Medical grade, Airgas, Radnor, PA) into a clathrate (cage) molecule, 2-hydroxypropyl)-β-cyclodextrin (HPCD; Sigma-Aldrich, St. Louis, MO). HPCD (2.5 mg) was incubated with Xe gas under 3 atm pressure at −80 °C for 3 days. A Xe-saturated water solution (5 ml) was made by incubating Xe gas in water under 3 atm at 4 °C for 3 days. The Xe-enriched solution was made by mixing Xe-HPCD complex (2.5 mg) with the Xe-saturated water solution.

### Measurement of Xe concentration

Xe concentration was measured using GC-MS (HP 6890-HP 5973A) interfaced with a G1512 controller, a GC injector (tower and tray), and an Edwards E2M2 rough pump (Agilent Technologies, Santa Clara, CA, USA)] as previously described^37^.

### Evaluation of Xe absorbance by oral administration

Absorbance of Xe was investigated in the WT C57BL/6J mice. Animals were fed with Xe-enriched solution (200 µl administrated by gavage), and blood was taken from the heart 10 minutes after administration of Xe-enriched solution. Xe concentration was measured by GC-MS as previously described^37^.

### Animal studies

In this study, ApoE gene KO mice were used. ApoE plays important roles in the development of hypercholesterolemia and atherosclerotic lesions. ApoE gene KO mice display poor lipoprotein clearance with subsequent accumulation of cholesterol ester-enriched particles in the blood, which promote the development of atherosclerotic plaques. It is widely used for studying the pathogenesis of cardiovascular disease including atherosclerosis.

All animal studies were approved by the Animal Welfare Committee at the University of Texas Health Science Center at Houston. All experiments were performed in accordance with relevant guidelines and regulations. WT C57BL/6J mice and ApoE KO transgenic mice^38^ were obtained from Jackson Laboratory (Bar Harbor, ME). The WT C57BL/6J mice were used as controls. Six- to 10-month-old male controls and ApoE KO mice were divided into five groups: 1) WT controls fed normal diet; 2) ApoE KO mice fed normal diet; 3) ApoE KO mice fed high-fat diet (60% calories from fat, Envigo TD 06414, Houston, TX); 4) ApoE KO mice fed high-fat diet + vehicle-only (HPCD-only solution, 200 µl administrated by gavage, once a day); and 5) ApoE KO mice fed high-fat diet + Xe-enriched solution (200 µl administrated by gavage, once a day). After 6 weeks, cardiovascular function and blood pressure were assessed. Mice were euthanized and tissue samples taken (Fig. 1).

### Echocardiography and electrocardiography

Serial M-mode echocardiography was conducted to evaluate alterations in cardiac morphology and cardiac function using a Vevo 770 Imaging System (VisualSonics Inc., Ontario, Canada) equipped with a 30-MHz microprobe]. M-mode echocardiography measurements were taken at baseline prior to treatment and immediately after 6 weeks of treatment for each group. Heart rate, LVIDd, LVPWd, IVSd, FS, EF, CO, LV volume, and LV mass corrected to body surface area were measured from echocardiographic data. Heart weight and body weight were obtained after 6 weeks of treatment, and the heart to body weight ratio presented by the number of milligrams of heart tissue for every gram of body-weight.

### Blood pressure measurements

Systolic and diastolic blood pressures were measured on conscious mice using a non-invasive computerized automated tail-cuff system (Vistech BP-2000 Blood Pressure Analysis System; Vistech Systems, Apex, NC)^39^.

### High-performance liquid chromatography analysis of tetrahydrobiopterin

Heart BH4 and BH2 contents were measured by HPLC^40^. Briefly, the heart tissue was quickly weighed and snap-frozen at −180 °C. Lysis buffer (1 ml) containing 3.8% perchloric acid, 1 mg/ml dithioerythritol (DTE), and 1 mg/ml diethylenetriaminepentaacetic acid (DTPA) was added into the frozen tissue (80 mg). Tissue was homogenized on ice by drill pressing (3–5 passes) and the homogenate centrifuged at 13,200 rpm for 10 minutes at 4 °C. The supernatant was collected and analyzed using a Waters 2695 HPLC pump (Milford, MA) interfaced with an ESA Coularray four-channel electrochemical detector (Chelmsford, MA). Separation was conducted using a 250 × 4.6-mm inner diameter Ultrasphere reversed-phase C18 column (Beckman Coulter, Fullerton, CA) and isocratic elution performed with 5% methanol and 95% 83 mM sodium acetate containing 5.5 mM citric acid, 54 µM ethylenediaminetetraacetic acid (EDTA), and 160 µM dithioerythritol (DTE) at a flow rate of 0.4 ml/min. Electrochemical detector channels were set to 0, 150, 365, and 550 mV. BH4 was quantitated by addition of peak areas measured on the latter three channels, and BH2 quantitated on the combined latter two channels. Solutions of ultrapure BH4 and BH2 (Cayman Chemical, Ann Arbor, MI) freshly prepared in 0.1 N perchloric acid containing 1 mg/ml DTPA and 1 mg/m DTE were analyzed. Collected data were normalized to tissue weight and converted to the ratio of BH4 to BH2.

### Protein concentration

Fresh frozen heart tissue (100 mg) was thawed, placed in 1 ml of radio-immunoprecipitation assay buffer (Cell Signaling Technology, Inc. Danvers, MA) containing protease inhibitors (complete protease inhibitor cocktail, Sigma), and homogenized by sonicating for 20 seconds on ice. The homogenate was centrifuged for 10 minutes at 14,000 g at 4 °C. The supernatant was utilized to determine protein concentration using a Bradford Protein Assay kit (Bio-Rad, Hercules, CA) kit.

### Creatine kinase-MB isoenzyme (CKMB) measurements

Plasma CK-MB was measured using the CK-MB ELISA Kit (TSZELISA, Waltham, MA). Tissue and plasma samples were placed in 96-well microtiter plates and incubated for 30 minutes at 37 °C. After buffer washing 4 times, CK-MB antibodies were added to each well and incubated 2–3 hours at room temperature. Fluorescent intensity was measured using a SpectroMax Microplate reader (Bio-Tek Instruments, Winooski, VT) at 450 nm. All samples were prepared and analyzed in triplicate.

### Isolation and hypoxic culture of primary ventricular myocytes

Neonatal (within 24 hours after birth) mouse ventricular myocytes were isolated and cultured^41^. Neonatal mice (n = 12) were euthanized and hearts harvested and placed in ice-cold Hanks’ solution containing 5 mM HEPES (pH 7.4) and 100 U/ml penicillin-streptomycin (ThermoFisher Scientific, Waltham, MA). After the atria were removed, the ventricles were sliced into small pieces (2–4 pieces per heart) and washed 3 times using cold fresh Hanks’ solution. The tissue was digested using fresh Hanks’ solution containing 1 mg/ml collagenase type II (Worthington Biochemicals. Lakewood, NJ) and subjected to gentle rocking for 2 hours at room temperature. Suspended cells were pelleted by centrifugation at 800 g for 5 minutes at 4 °C. To enrich the cardiomyocytes, the dissociated cells were cultured in medium with Dulbecco’s Modified Eagle’s Medium (DMEM; Invitrogen, Life Technologies) containing 10% fetal bovine serum (Invitrogen, Life Technologies), L-glutamine (2 mM, Invitrogen, Life Technologies), 5 mM HEPES (pH 7.4), and 100 U/ml penicillin-streptomycin (Invitrogen, Life Technologies) in a humidified atmosphere with 5% CO_2_ for 1 hour at 37 °C. This procedure allows non-cardiomyocytes to attach to the bottom of the culture dishes. The resultant suspension containing cardiomyocytes was transferred into 6-well plates and cultured in a humidified atmosphere and 5% CO_2_ at 37 °C. Once confluent monolayers were formed in the cultured cardiomyocytes, cells were rendered quiescent by culture in serum-free DMEM for 48 hours. The quiescent cells were treated with or without 10 µL of Xe-enriched solution and incubated with DMEM without fetal bovine serum in a hypoxic incubator (0.5% O_2_, 5% CO_2_, 37 °C) for 24 hours. Cells were lysed with cell lysis buffer (Cell Signaling Technology, Inc., Danvers, MA) and subjected to Western Blot analysis.

### Western blot analysis

Studies have shown that BDNF is involved in Xe activity and molecular mechanisms of heart disease^38^. We hypothesized that Xe affects cardiac function via BDNF-mediated signal pathways. To test this hypothesis, cardiac BDNF expression was measured using ELISA and Western Blotting. In addition, cTnI is highly related to myocardial disfunction. We also measured cTnI expression in myocardial tissue. Total- and phospho-HIF-1α, BDNF, phospho-PKC, total- and phospho-Akt, total- and phospho-PKA, total and phospho-EKR1/2 were measured by Western Blot^42^. For immunoblot analysis, lysed tissue or cellular proteins were separated using sodium dodecyl sulphate-polyacrylamide gel electrophoresis (SDS-PAGE, 4–12%) gradient gels, and transferred to polyvinylidene fluoride membranes (Millipore Corporation, Billerica, MA). Blots were incubated overnight at 4 °C with primary antibodies, washed three times with Tris-buffered saline containing 0.1% Tween 20, and probed with secondary antibodies (LI-COR Biosciences, Lincoln, NE). Blots were stripped and re-probed with other antibodies. Densitometric analysis of the immunoblots was performed using an Odyssey Infrared Imager (LI-COR Biosciences, Lincoln, NE).

### Statistical analysis

Data were processed using Microsoft Excel and GraphPad Prism 5.0. All values are expressed as mean and standard error of the mean (SEM) with n = 5 in each group. For comparison between multiple groups, the Kruskal-Wallis analysis of variance (ANOVA) was performed followed by post hoc multiple comparisons of mean ranks for all groups. P-values less than 0.05 were considered significant.

## Data Availability

The datasets generated and/or analysed during the current study are available from the corresponding author on reasonable request.
